# Experiments upon the Supra-Renal Capsules

**Published:** 1857-05

**Authors:** 


*Experiments upon the Supra-renal Capsules.*
By M. Brown-Sequard and others.

Dr. Addison's investigations into the diseased condition of the renal capsules, have among other results had the effect of recalling attention to the functions of, and amount of importance to be attached to, these organs. As usual, in France these questions have been sought to be settled by multiplied vivisections; and already numerous communications detailing the results of experiments upon animals have been laid before the Academy of Sciences, the reports of such results being somewhat at variance with each other.

M. Gratiolet states that his experiments upon guinea-pigs go back to 1853–4, and the present discussions have induced him to publish the results. He found that when the left capsule alone was removed that neither death nor convulsions necessarily followed; but when the right and, *à fortiori*, when both were removed death always took place. He believes this arises from the anatomical relations of the right capsule with the liver and vena cava inferior, which render the operation as dangerous as it is difficult.

M. Phillipeaux reports that extirpation of both capsules in the albino rat does not necessarily cause death; for in four of these animals in whom he successfully extirpated both, no important functional disturbances resulted. He believes that when death has resulted it has done so from the operation itself, which is a severe one, and may be followed by fatal inflammation of the perineal cellular tissue, peritonitis, or intestinal hernia through the divided muscles.

In connection with this communication we may notice another made to the Academy by Dr. Martini, who relates the case of a man dying of phthisis at the Hospital for Incurables at Naples. The two kidneys, instead of occupying their normal position, were found fused into a single body, lying on the promontory of the sacrum. This body received a single emulgent artery, which divided into four branches, having corresponding veins also uniting into a single emulgent vein. Two very short ureters of the usual calibre proceeded to the bladder. The structure of the kidney was normal. Not a trace of renal capsules could be discovered, although, as the author was aware of Addison's researches, he made careful search for them.

M. Brown Séquard's communication, since expanded by him, enters into minute detail. After taking a review of the state of science in regard to these bodies prior to Addison's researches, he details the results of his experiments. All the animals in which he has extirpated, crushed, or even pricked at several points both capsules, have died soon after the operation. Of 51 rabbits in which the precise time of death was noted, the mean period of survival was found to be nine hours and some minutes; the minimum, five hours and a half, and the maximum (in 1 only,) fourteen hours and a half. In dogs and adult cats, the survival was somewhat longer, the mean period being fourteen hours, while in the guinea-pigs it was thirteen hours. He thinks the longer period of survival observed by Gratiolet was due to his employing young animals, in which the survival is longer; and in his own experiments he found that in very young cats and dogs its mean rose to thirty-seven hours. The mean period of survival in the 90 experiments performed on the various animals was seventeen hours and a half, the mean for adult animals being about twelve hours, for very young animals, thirty hours. When only one capsule is removed or injured the duration of life is somewhat longer, but, although death in this case is not inevitable, it is very frequent, inasmuch as only 2 out of 37 animals survived. The supposition of Gratiolet, that abstraction of the right capsule is always fatal, is not borne out by a more extended observation.

Having detailed these results, M. Brown-Séquard next proceeds to give a description and explanation of the phenomena produced:

*Influence exerted on animal and organic life.*—1. There is a notable debility produced, differing from that merely due to the pain of the operation, which is temporary. It gradually increases, and becomes excessive as death approaches. 2. The respiration and circulation undergo important modifications. The respiration may be temporarily increased in frequency while the beats of the heart are diminished. At a somewhat more advanced period, the reverse of these conditions prevails; while, as the case proceeds, the frequency of both diminish. In the immense majority, the force of the heart's pulsations are notably diminished during the whole period of survival, and especially towards its latter part. 3. The appetite quite disappears; and, if the animal has eaten before, digestion is arrested, but there is rarely vomiting. 4. The urinary secretion remains normal. 5. The temperature generally becomes diminished, and especially in winter, when rabbits towards the end will lose 4° or 5° C. in a room at 10° or 12° C. 6. Sensibility persists to the last, and even seems exaggerated. 7. Delirium and convulsions are among the principal phenomena, the latter being sometimes very violent, and manifesting themselves either under the tetanic or epileptic form.

*Nature of the ensuing death.* Death takes place sometimes from asphyxia, sometimes from syncope; and the asphyxia may take place either suddenly or gradually. Not unfrequently, some time before death, in place of convulsions, a turning movement is set up, or the animal rolls alternately to the left and right, as after certain lesions of the encephalon.

*Pigmentary matter and peculiar crystals found in the blood.* The coëxistence of the deposit of pigment in the skin and changes in the capsules in the human subject, gives much interest to the fact of the author having found a notable increase of the normal pigmentary matter in the blood of the animals operated upon. No increase of pigment has been found in the skin of these animals. Blood collected from animals who have undergone the ablations, exhibits the rapid spontaneous formation of crystals. They are especially met with in the blood of the inferior vena cava, and differ in character from hæmatoidine.

*The cause of death.* In this section the author passes various possible causes of death under review, such as traumatic peritonitis, hæmorrhage, lesions of the liver or kidney, phlebitis; and comes to the conclusion, that this speedy death is not the result of any of these, or even their combination, and shows by numerous comparative experiments, that survival from these causes of death would be more prolonged. In like manner, though he believes that injury to the filaments coming from the semilunar ganglion may coöperate in producing the fatal results, these are not due to it alone; for great injury done to the portions of the sympathetic, whence the supply for the capsules is derived, is followed by a longer survival than in the case in question. As death cannot be explained by any of these causes, we arrive, by exclusion, at the ablation of the capsules, an inference rendered more probable by certain facts. When death follows the removal of only one capsule, the convulsions that ensue are generally stronger on one side, and the rolling movement, which is frequently observed, almost always commences on the opposite side. Not unfrequently, in rabbits, the pupil corresponding to the side on which the ablation has been made, is more contracted than the other.

*The pigmentary disease in rabbits.* During the last twelve years, M. Brown-Séquard has observed a very large number of rabbits die of a disease characterized by peculiar symptoms. For a long time he attributed its production to the presence of the ova of a species of helminthus, so constantly found in the liver of the Parisian rabbits, that he has never failed of finding them in 500 rabbits he has examined. The intensity of the disease seemed to be proportionate to the amount of destruction of the liver produced; while in the rabbits in America, in which these ova are not met with, the disease is also unknown. Still further observation has not always shown this coincidence between this state of the liver and the production of the disease; and the author's attention having been directed by Addison's researches to the condition of the renal capsules, he has found remarkable alterations in these bodies, some of which seem to be constant. The cortical substance, from a canary color becomes of a chocolate color, or of a more or less deep reddish brown, a *ramollissementt* of the substance, and a dilation of the capillaries being also observed. Such changes were observed in 26 out of 28 rabbits suffering from pigmentary disease. The symptoms observed have a marked resemblance with the phenomenon produced by the ablation of the capsules. In these animals there is accumulation of pigmentary matter in the blood. This, in the author's opinion, has induced irritation of the capsules, the function of which in the normal state consists in producing modifications in this pigmentary matter. The function of these glands being thus impeded, a still greater accumulation of pigmentary matter ensues, and death rapidly takes place in consequence, among other causes, of the obstacles which the pigmentary masses present to the free circulation of the blood in the capillaries of the nervous centres.

*Results of the experiments and the pigmentary disease compared with Addison's disease.* M. Brown-Séquard believes that the facts published by Addison and Hutchinson receive powerful illustration from the above experimental and pathological observations. Among the symptoms observed in the human subject there are two that have always been found, an impairment of voluntary motion and a discoloration of some part of the skin. In animals the debility is very remarkable; and if their death is too rapid to allow of the pigment being deposited in the skin it is found in the blood. A disordered state of the nervous system and loss of appetite are other symptoms often common to all these categories of facts.

*Functions of the supra-renal capsules.* Prior to Addison's observations, all that physiologists had established with regard to these bodies was, that they exerted a modifying power upon the blood, and differed from glands having ducts, inasmuch as they elimated nothing. Addison's observations, and the author's experiments, lead to the conclusion that these organs are essential to life. Indeed they seem more so than do the kidneys themselves, survival being longer after ablation of the latter. It is extremely probable that one of the functions of the capsules consists in the special modification of an unknown matter capable of transformation into pigment, and that the modification exerted on this matter by the capsules prevents such transformation. This would not seem to be the only function they exercise, for the spontaneous production of crystals in the blood of animals deprived of the capsules, and the prompt disappearance of globules, shows the great alteration this fluid undergoes. Other experiments made by the author, and hereafter to be related, show that the blood of animals deprived of the capsules often acts as a fatal poison when injected into the veins of animals of the same species.—*Med. Times and Gaz.*, Jan. 10, 1857, from *Archives Gén. de Med.*, Oct. and Nov., 1856; *Comptes Rendus*, Tome XLIII, Nos. 8, 9, 19, and 22.

